# HBx regulates fatty acid oxidation to promote hepatocellular carcinoma survival during metabolic stress

**DOI:** 10.18632/oncotarget.6817

**Published:** 2016-01-04

**Authors:** Ming-Da Wang, Han Wu, Shuai Huang, Hui-Lu Zhang, Chen-Jie Qin, Ling-Hao Zhao, Gong-Bo Fu, Xu Zhou, Xian-Ming Wang, Liang Tang, Wen Wen, Wen Yang, Shan-Hua Tang, Dan Cao, Lin-Na Guo, Min Zeng, Meng-Chao Wu, He-Xin Yan, Hong-Yang Wang

**Affiliations:** ^1^ International Cooperation Laboratory on Signal Transduction, Eastern Hepatobiliary Surgery Institute, The Second Military Medical University, Shanghai 200438, P.R. China; ^2^ National Center for Liver Cancer Research, Shanghai 201805, P.R. China; ^3^ Department of Surgery, Eastern Hepatobiliary Surgery Hospital, Second Military Medical University, Shanghai 200433, P.R. China; ^4^ State Key Laboratory of Oncogenes and Related Genes, Shanghai Cancer Institute, Renji Hospital, Shanghai Jiao Tong University, Shanghai 200032, P.R. China

**Keywords:** hepatitis B virus X protein, fatty acid oxidation, energy homeostasis, metabolic stress, cell survival

## Abstract

Due to a high rate of nutrient consumption and inadequate vascularization, hepatocellular carcinoma (HCC) cells constantly undergo metabolic stress during tumor development. Hepatitis B virus (HBV) X protein (HBx) has been implicated in the pathogenesis of HBV-induced HCC. In this study, we investigated the functional roles of HBx in HCC adaptation to metabolic stress. Up-regulation of HBx increased the intracellular ATP and NADPH generation, and induced the resistance to glucose deprivation, whereas depletion of HBx via siRNA abolished these effects and conferred HCC cells sensitive to glucose restriction. Though HBx did not affect the glycolysis and oxidative phosphorylation capacity of HCC cells under normal culture conditions, it facilitated fatty acid oxidation (FAO) in the absence of glucose, which maintained NADPH and ATP levels. Further investigation showed that HBx expression, under glucose deprivation, stimulated phosphorylation of AMP-activated protein kinase (AMPK) and acetyl-CoA carboxylase (ACC) via a calcium/CaMKK-dependent pathway, which was required for the activation of FAO. Conversely, inhibition of FAO by etomoxir (ETO) restored the sensitivity of HBx-expressing cells to glucose deficiency *in vitro* and retarded xenograft tumor formation *in vivo*. Finally, HBx-induced activation of the AMPK and FAO pathways were also observed in xenograft tumors and HBV-associated HCC specimens. Our data suggest that HBx plays a key role in the maintenance of redox and energy homeostasis by activating FAO, which is critical for HCC cell survival under conditions of metabolic stress and might be exploited for therapeutic benefit.

## INTRODUCTION

Hepatocellular carcinoma (HCC) is among the top five most frequent cancers and the third leading cause of cancer death worldwide [1]. Chronic infection with hepatitis B virus (HBV) is a major risk factor for HCC [2]. Hepatitis B virus X protein (HBx), which encodes a small 17-kDa soluble protein, is one of four overlapping open reading frames (ORFs) in the HBV genome and has been involved in the development of HBV-associated HCC [3]. Though HBx possesses transcriptional regulatory properties and modulates cell cycle progress, apoptosis, protein degradation, and genetic stability by directly or indirectly interacting with a variety of intracellular proteins [4-7], its role in the regulation of cellular metabolism during hepatocarcinogenesis has yet to be elucidated.

The activation of oncogenes or loss of tumor suppressors contributes to altered cellular metabolism [8, 9], which has emerged as a hallmark of cancer [10], and plays an important role in cancer pathogenesis and development [11]. It is widely acknowledged that the Warburg effect, with characteristics of enhanced glucose uptake and lactate production, is one of the most important metabolic transformations accompanying tumorigenesis [12]. In addition, increased glutamine metabolism and aberrant fatty acid metabolism are also frequently observed in cancer cells [13]. Therefore, a better understanding of these metabolic adaptations may warrant further exploitation for potential therapeutic benefits [14, 15].

Due to high rate of glycolytic flux and inadequate vascularization, solid tumors usually grow under conditions constantly depleted of oxygen and crucial nutrients, particularly glucose [16, 17]. In this scenario, cancer cells must adapt their metabolic profiles to unfavorable microenvironments and exhibit different metabolic dependencies compared to their normal counterparts [18]. For instance, tumor cells may shift their metabolic phenotypes from glycolysis to fatty acid oxidation (FAO; also known as β-oxidation) for the purpose of maintaining cell survival under such stressful conditions [19, 20]. Likewise, the increased FAO was required for tumor cells to produce ATP and resist nutrient deficiency [21]. Moreover, the relevance of FAO for cell survival after loss of attachment to the extracellular matrix was also observed in breast cancer [22]. Concrete evidence has demonstrated that nutrient deprivation enhances fatty acid synthesis and induces lipid droplet (LD) biogenesis, which further fuels lipid oxidation to maintain energy homeostasis and promote cell survival [23]. HBx was shown to accelerate lipogenesis and caused hepatic steatosis via transcriptional activation of metabolism-related genes or mediating a series of signaling pathways [24-27]. Despite the established relationship between HBx and lipid metabolism in HCC pathogenesis, the precise roles of HBx in HCC cells experiencing metabolic stress remain unknown.

In present study, we report that HBx promotes HCC survival by inducing lipid oxidation to maintain NADPH and ATP homeostasis in the absence of glucose, thus providing new insights into the HBx-mediated metabolic transformation during hepatocarcinogenesis. Our findings also establish a novel function of HBx in sustaining HCC survival during metabolic stress and suggest that targeting FAO may benefit the treatment of HBV-related HCC.

## RESULTS

### HBx expression confers a survival advantage to HCC cells during glucose deprivation

**Figure 1:**
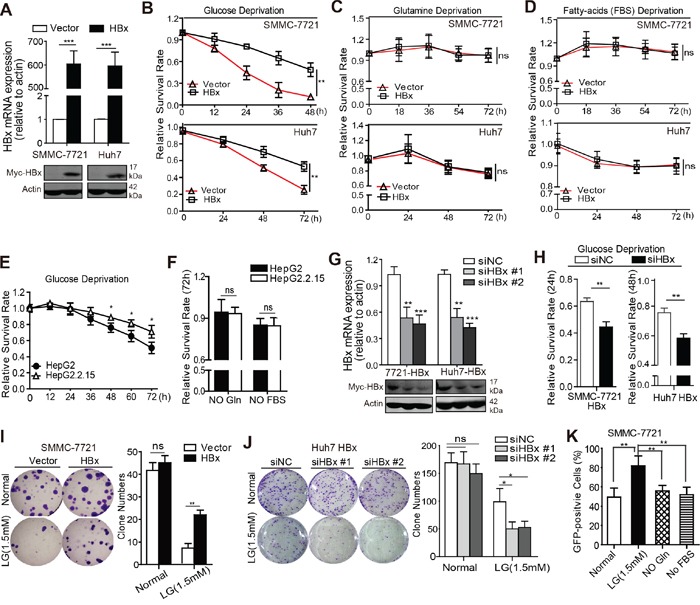
HBx expression confers a survival advantage to HCC cells during glucose deprivation **A.** qPCR and immunoblotting analyses of exogenous HBx expression in SMMC-7721 and Huh7 cells transfected with vector or myc-tagged HBx. **B.** Relative cell survival rates of SMMC-7721 and Huh7 cells stably expressing empty vector or HBx in the absence of glucose (−Glc) were measured at indicated time points. HBx expression promoted HCC cells survival under glucose depletion (−Glc) (**p<0.01). **C-D.** HBx expression had no significant effect on cell survival under conditions of glutamine (−Gln) or FBS deprivation (−FBS). **E.** Relative cell survival rates of HepG2 and HepG2.2.15 cells in the absence of glucose (−Glc) were measured at indicated time points (*p<0.05). **F.** HepG2 and HepG2.2.15 cells were maintained under glutamine deprivation (−Gln) or FBS withdrawal (−FBS), respectively, and cell survival rates were detected at 72h. **G.** siRNAs (Sequence #1 and #2) targeting HBx reduced the mRNA and protein levels of HBx in SMMC-7721-HBx and Huh7-HBx cells (**p<0.01; ***p<0.001). **H.** Depletion of HBx by siRNA sensitized SMMC-7721-HBx and Huh7-HBx cells to glucose deprivation-induced cell death (**p<0.01). All the values in B-F and H were expressed as the fold change relative to their corresponding untreated controls (presented as equal to 1) at the onset of the assays. **I-J.** Expression of HBx enhanced the colony formation capacity of SMMC-7721 cells (n=500) under low glucose condition (1.5mM) but inhibition of HBx by siRNA (#1 and #2) had opposite effect in Huh7 cells stably expressing HBx. Colony numbers (mean±SD) from three independent experiments and representative results were shown (**p<0.01). **K.** For *in vitro* competition assay, HBx expression conferred a competitive growth advantage to SMMC-7721 cells under glucose limitation *in vitro*. Experiments were performed in triplicate and data are shown as mean ± SD (**p<0.01).

To investigate the effect of HBx on HCC cell survival during metabolic stress, HCC cell lines SMMC-7721 and Huh7 were transfected with HBx or a vector control, and the expression of exogenous HBx was verified by quantitative RT-PCR and immunoblot analysis (Fig. 1A). Next, we cultured SMMC-7721 and Huh7 cells in medium deprived of glucose, glutamine, or fetal bovine serum (FBS) to mimic different metabolic stress conditions. Notably, compared with control cells, HBx-expressing cells were more resistant to cell death induced by glucose depletion (Fig. 1B), whereas no significant protective effect was observed in SMMC-7721 or Huh7 cells under conditions of glutamine or serum deprivation (Fig. 1C, 1D). We further evaluated the pro-survival effect of HBx under glucose deprivation in HBV-infected HepG2.2.15 cells. As expected, HepG2.2.15 cells were more resistant to glucose deprivation but showed no significant survival advantage under conditions of glutamine or FBS depletion in comparison with HepG2 cells (Fig. 1E, 1F). Consistently, knockdown of HBx in HBx-expressing cells accelerated cell death induced by glucose limitation (Fig. 1G, 1H), but did not affect cell viability under glutamine or FBS deprivation (Supplementary Fig. 1A, 1B). In addition, overexpression of HBx enhanced colony formation in low-glucose medium, while depletion of HBx by HBx siRNA had the opposite effect (Fig. 1I, 1J). We next assessed the effect of HBx on cell survival in low glucose using an *in vitro* competition assay. SMMC-7721 cells co-expressing green fluorescent protein (GFP) and HBx or control plasmids were mixed 1:1 and maintained in the presence or absence of glucose, glutamine or FBS for 4 days. The percentages of GFP^+^ subsets were determined by flow cytometry. As expected, HBx-expressing cells displayed a competitive growth advantage under glucose limitation *in vitro* compared to cells expressing the control vector (Fig. 1K). Taken together, these data indicate that HBx provides HCC cells with a survival advantage during glucose deprivation.

### HBx maintains intracellular redox and energy homeostasis during glucose deprivation

**Figure 2:**
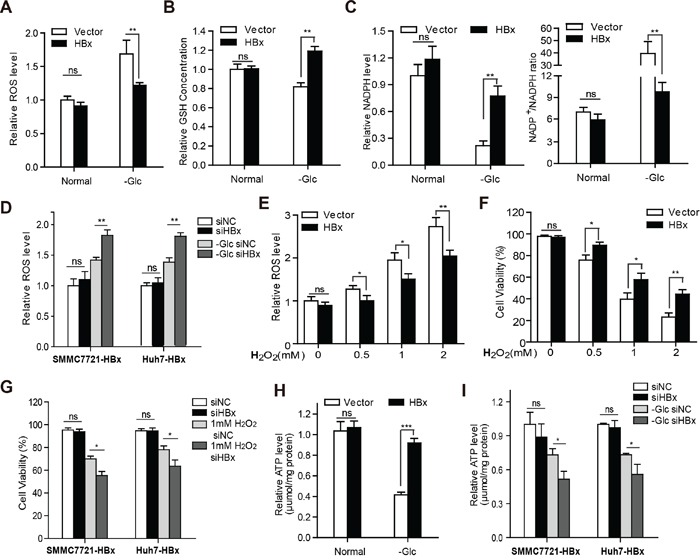
HBx maintains intracellular redox and energy homeostasis during glucose deprivation **A.** Overexpression of HBx reduced the production of intracellular ROS level under glucose deprivation. **B.** HBx stabilized intracellular GSH level, and maintained NADPH content and NADP+/NADPH ratio **C.** upon glucose limitation. **D.** Knockdown of HBx by siRNA increased the ROS level in SMMC-7721-HBx and Huh7-HBx cells when exposed to the lack of glucose. **E-F.** SMMC-7721 cells stably expressing vector or HBx were stimulated with various concentrations of H_2_O_2_ for 12h. The intracellular ROS content and cell viability were detected. **G.** Downregulation of HBx by siRNA sensitized SMMC-7721-HBx and Huh7-HBx cells to cell death when exposed to H_2_O_2_ stimulation. **H.** The intracellular ATP levels of SMMC-7721 vector or HBx cells cultured in the presence or absence of glucose were measured. **I.** Knockdown of HBx by siRNA attenuated the ATP production in SMMC-7721-HBx and Huh7-HBx cells under glucose deprivation. Experiments were performed in triplicate and data were shown as mean ± SD. Results in A-E, and H, I were expressed as the fold change relative to their corresponding untreated controls (presented as equal to 1). (*p<0.05; **p<0.01; ***p<0.001).

Glucose depletion could induce cell death by causing oxidative stress and disturbing energy homeostasis. We thus investigated whether HBx influences the intracellular redox balance and energy supply under glucose limitation. The intracellular reactive oxygen species (ROS) level was examined by employing the redox-sensitive probe CellROX. Remarkably, HBx expression significantly prevented the increase in ROS levels (Fig. 2A) and maintained GSH content and the NADP+/NADPH ratio in the absence of glucose (Fig. 2B and 2C), while silencing of HBx abolished this effect (Fig. 2D). Moreover, overexpression of HBx enhanced the elimination of H_2_O_2_ or hypoxia-induced ROS (Fig. 2E and Supplementary Fig. 2A) in hepatoma cells. Consistently, up-regulation of HBx in hepatoma cells provided resistance to H_2_O_2_ or hypoxia-induced cell death (Fig. 2F and Supplementary Fig. 2B), whereas knockdown of HBx sensitized hepatoma cells expressing HBx to oxidative stress-induced cell death (Fig. 2G). These results indicate that HBx protects HCC cells from metabolic stress by maintaining redox equilibrium. As ATP content is indicative of cellular energy level, we next determined the ATP level and found that HBx expression partially reversed ATP depletion under glucose withdrawal (Fig. 2H), while HBx knockdown abolished the effect (Fig. 2I). Together, these results show that HBx promotes HCC survival in the absence of glucose via the generation of reducing equivalents and elevation of ATP contents, thus maintaining intracellular redox and energy homeostasis.

### HBx does not affect the glycolysis and oxidative phosphorylation capacity of HCC cells

**Figure 3:**
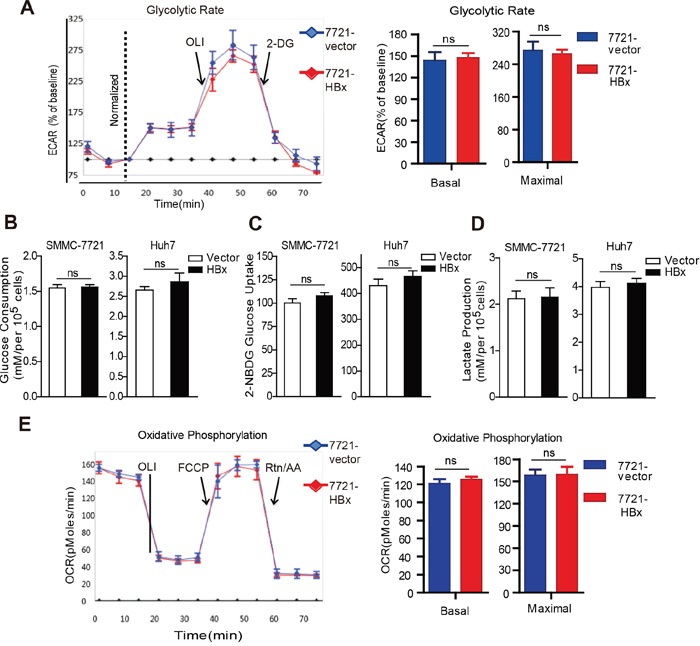
HBx does not affect the glycolysis and oxidative phosphorylation capacity of HCC cells **A.** The effect of HBx on glycolysis in control or HBx-expressing cells was evaluated by monitoring extracellular acidification rate (ECAR) using XF96 Extracellular Flux Analyzer. Basal and reserved glycolytic capacity was measured with or without mitochondrial inhibitor oligomycin (OLI, 1mg/ml) and the glycolysis inhibitor 2-DG (5mM). All the values were expressed as the percentage of baseline ECAR. **B.** Under standard cultural condition, HBx expression had little effect on glucose consumption and glucose uptake following the incubation of 2-NBDG **C**. **D.** HBx expression had no significant effect on lactate production. All the results were normalized to cell number. **E.** Basal and maximal oxygen comsuption rate (OCR) in control or HBx cells was determined via Seahorse XF96 extracellular flux analyzer. Experiments were performed in triplicate and data are shown as mean ± SD.

We next determined the effect of HBx on cellular metabolism and energy production in HCC cells. The Seahorse XF96 Extracellular Flux Analyzer was used to evaluate the metabolic dependence of HBx-expressing cells on the two major metabolic processes: glycolysis and mitochondrial oxidative phosphorylation (OXPHOS). As shown in Fig. 3A, expression of HBx had no significant impact on the extracellular acidification rate (ECAR), glucose consumption, glucose uptake, or lactate production (Fig. 3B-3D and Supplementary Fig. 3A-3C). Moreover, SMMC-7721 cells expressing HBx also displayed little difference in basal and maximal oxygen consumption rate (OCR) (Fig. 3E), indicative of similar oxidative metabolism and reserve respiration capacity, as compared to control cells. Collectively, our metabolic analyses suggest that HBx expression does not cause a metabolic switch to either OXPHOS or glycolysis to meet the energy demand and maintain intracellular redox homeostasis under normal cultural conditions.

### HBx promotes FAO in an AMPK-dependent manner in the absence of glucose

**Figure 4:**
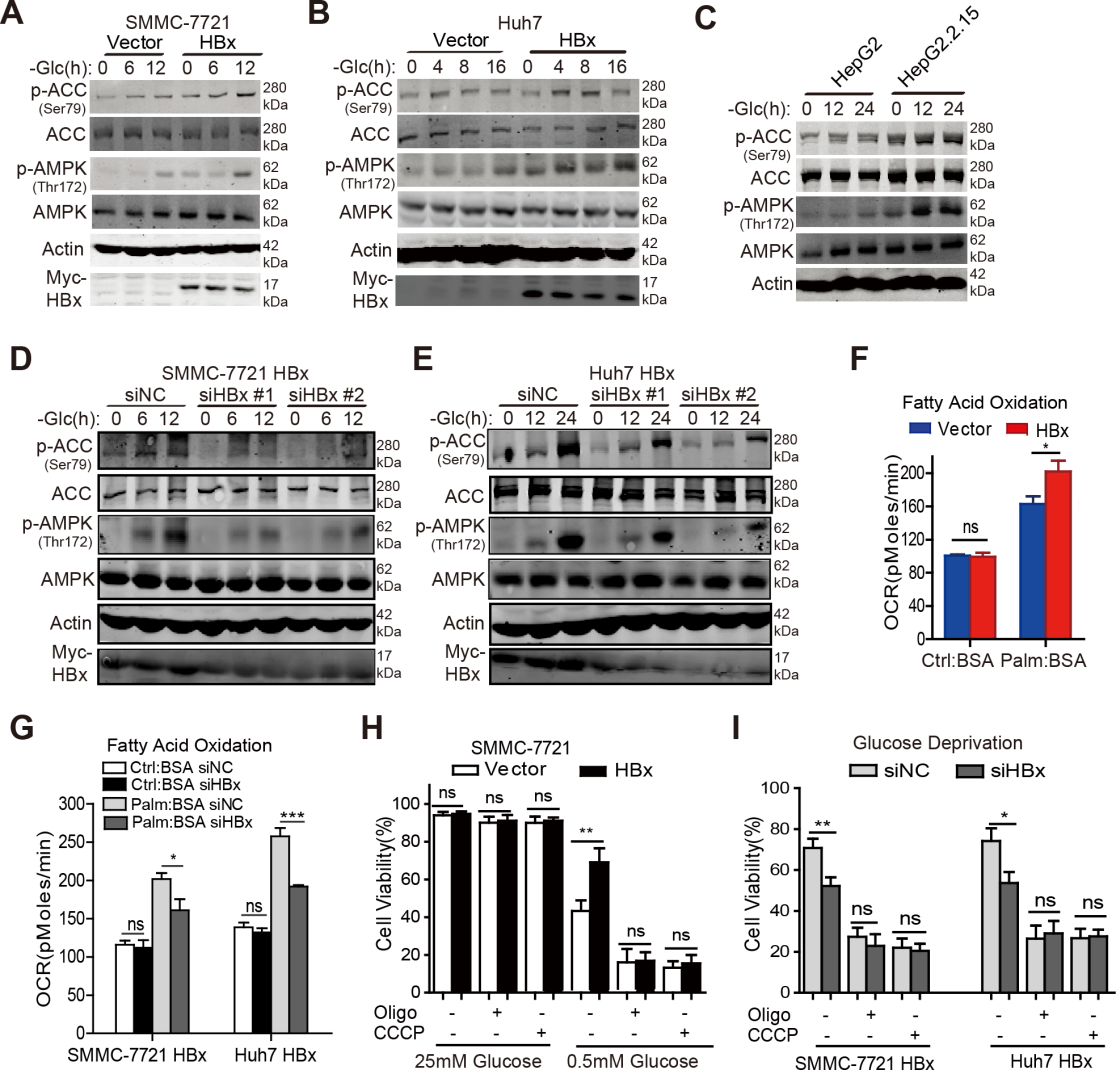
HBx promotes FAO in an AMPK-dependent manner in the absence of glucose **A-C.** Immunoblotting analysis of p-ACC (Ser79), ACC, p-AMPK (Thr172), and AMPK in indicated hepatoma cells cultured in the presence or absence of glucose at the indicated time points, β-actin was used as internal loading control. **D-E.** Downregulation of HBx by siRNAs (#1 and #2) attenuated the phosphorylation level of AMPK and ACC in SMMC-7721-HBx and Huh7-HBx cells under glucose deprivation. β-actin was used as internal loading control. **F.** HBx-expressing cells exhibited enhanced fatty acids β-oxidation capacity compared with control cells under nutrient starvation. The OCR in pmol/min indicative of fatty acid oxidation (FAO) capacity was monitored and cells treated with BSA were served as a negative control. (*p<0.05). **G.** Knockdown of HBx by siRNA weakened the oxidation of exogenous fatty acids when cells were starved. (*p<0.05; ***p<0.001). **H.** Control or HBx-expressing HCC cells were treated with CCCP (5μM) or oligomycin (1μM) for 24h in complete (25mM) or low-glucose (0.5mM) medium and the cell viabilities were determined. **I.** SMMC-7721-HBx and Huh7-HBx cells transfected with siRNA targeting HBx or negative control were cultured as mentioned above and the cell viability was detected. Experiments were performed in triplicate and data were shown as mean ± SD. (*p<0.05; **p<0.01).

As AMP-activated protein kinase (AMPK) is a key metabolic sensor that promotes cell survival by regulating ATP homeostasis and NADPH maintenance in response to metabolic stress [19, 28], we determined whether HBx can activate the AMPK signaling pathway. Interestingly, overexpression of HBx induced the phosphorylation of AMPK and its downstream effector acetyl-CoA carboxylase (ACC) in SMMC-7721 and Huh7 cells under glucose depletion in a time-dependent manner (Fig. 4A, 4B). Similar results were observed in HepG2.2.15 cells (Fig. 4C). In contrast, downregulation of HBx hindered the phosphorylation of AMPK and ACC under glucose limitation (Fig. 4D, 4E). As activation of the AMPK pathway switches off anabolic processes and enhances catabolic processes, including lipid oxidation, we presumed that HBx might facilitate the FAO process to replenish NADPH and ATP levels during glucose depletion. In support of this notion, the β-oxidation capacity was significantly higher in HBx-expressing cells compared with control cells upon addition of exogenous palmitate during a period of starvation (Fig. 4F). Furthermore, knockdown of HBx attenuated the fueling of exogenous fatty acids under starvation (Fig. 4G). As fatty acids are finally transported into mitochondria for degradation via β-oxidation, we assessed whether inhibition of mitochondrial function would block the HBx-mediated pro-survival effect under metabolic stress. As expected, suppression of mitochondrial function by CCCP and oligomycin reversed the survival advantage conferred by HBx expression upon glucose depletion, whereas no obvious impact on cell survival was observed under normal conditions, even in the presence of CCCP or oligomycin (Fig. 4H, 4I). Taken together, these results suggest a critical role for HBx in promoting HCC cell survival by activating the AMPK and FAO pathways under glucose deprivation.

### HBx-induced FAO activation contributes to HCC cell survival after glucose withdrawal

**Figure 5:**
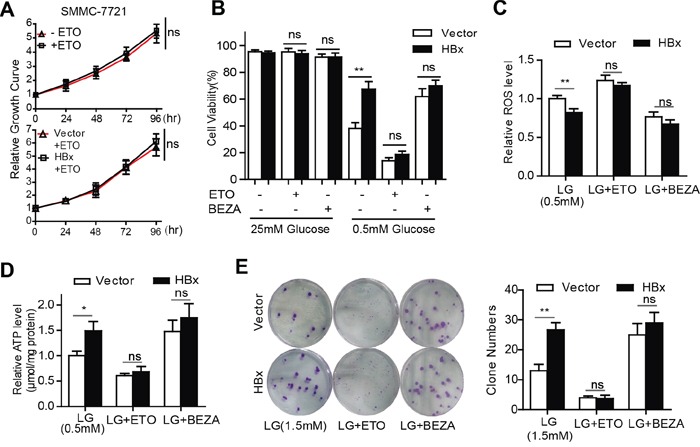
HBx-induced FAO activation contributes to HCC cell survival after glucose withdrawal **A.** Relative cell growth curves of SMMC-7721 cells maintained in the presence or absence of 100μM ETO (Up panel). For the control or HBx cells cultured in standard grown medium, 100μM ETO was added to the medium (low panel). **B.** Under glucose limitation (0.5mM), SMMC-7721 vector or HBx cells were treated with ETO (100μM), BEZA (400μM), or vehicle control for 24 hours and the cell viability was determined. (**p<0.01). **C-D.** Hepatoma cells expressing vector or HBx were cultured in low-glucose (0.5mM) medium in the presence of ETO (100μM), BEZA (400μM), or vehicle control for 24 hours. The intracellular ROS level and ATP content were assessed. (*p<0.05; **p<0.01). **E.** Under low-glucose (0.5mM) condition, ETO (100μM) treatment attenuated the colony-formation viability of HBx-expressing cells while treatment with BEZA (400μM) increased the colony numbers of both vector or HBx cells. Representative images from three independent experiments were shown and colony numbers (mean±SD) were countered. (**p<0.01).

Transport of long-chain fatty acids into mitochondria through carnitine palmitoyltransferase I (CPT-1) is the rate-limiting step in FAO. It is widely accepted that the FAO inhibitor etomoxir (ETO) prevents the entry of FAs into mitochondria by blocking the activity of CPT1. We noted that treatment with ETO did not impede cell growth under normal culture conditions (Fig. 5A). However, suppression of FAO by ETO markedly sensitized HBx-expressing cells to glucose limitation-induced cell death (Fig. 5B), accompanied by increased cellular ROS levels and ATP depletion (Fig. 5C, 5D). Conversely, activation of FAO by bezafibrate (BEZA), an activator of peroxisome proliferator activated receptor alpha (PPARα), prolonged cell survival upon glucose withdrawal (Fig. 5B), reduced the ROS content and enhanced intracellular ATP generation (Fig. 5C, 5D). Consistently, inhibition of FAO by ETO attenuated colony formation by HBx-expressing cells while BEZA treatment increased the colony numbers of control cells under low-glucose conditions (Fig. 5E). Taken together, these results suggest that HBx-mediated FAO is required to inhibit low-glucose-induced cell death by generating reductive NADPH and ATP, which are essential for the maintenance of energy and redox homeostasis.

### HBx induces AMPK activation via a cytosolic calcium/CaMKK-dependent pathway under glucose limitation

**Figure 6:**
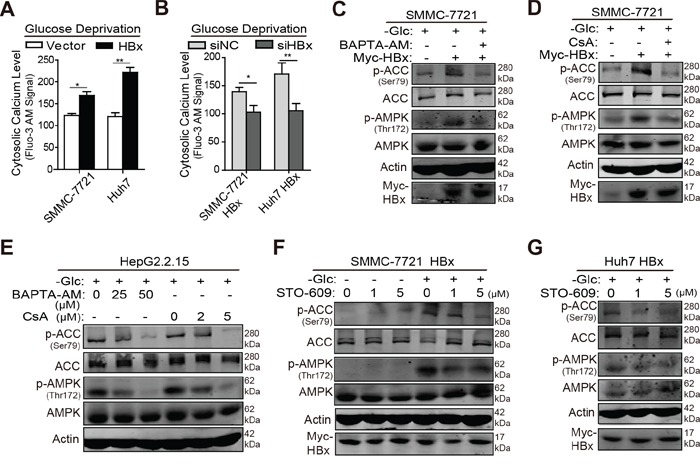
HBx induces AMPK activation via a cytosolic calcium/CaMKK dependent pathway under glucose limitation **A.** Hepatoma cells stably expressing vector or HBx were maintained in the medium without glucose for 8h and then treated with 5μM Fluo-3 AM for 45 min. The fluorescent signal was detected using flow cytometry. **B.** HBx-expressing cells transfected with siRNA targeting HBx or negative control were treated as mentioned above and the fluorescence intensity was measured. **C-D.** SMMC-7721 cells transfected with empty vector or HBx were incubated in glucose-free medium and treated with BATPA-AM (50μM) or CsA (5μM) for 8 hours. Then the cells were lysed to measure the phosphorylation states of AMPK and ACC by immunoblotting analysis. **E.** HepG2.2.15 cells were starved in glucose-free medium and treated with BATPA-AM (25μM, 50μM) or CsA (2μM, 5μM) for 16 hours. The phosphorylation states of AMPK and ACC were detected via immunoblotting analysis. **F.** SMMC-7721 HBx cells were treated with STO-609 (1μM, 5μM) in the presence or absence of glucose for 8 hours and the p-AMPK and p-ACC levels were detected by immunoblotting analysis. **G.** Huh7 HBx cells were maintained in glucose-free medium and treated with STO-609 (1μM, 5μM) for 16 hours, then the p-AMPK and p-ACC levels were detected by immunoblotting analysis. For all the western blot assays, β-actin was used as internal loading control. Experiments were performed in triplicate and data were shown as mean ± SD. (*p<0.05; **p<0.01).

Next, we explored the underlying mechanism by which HBx phosphorylates AMPK in the absence of glucose. A well-known upstream component of the AMPK pathway is calcium/calmodulin-dependent protein kinase kinases (CaMKKs), whose activity is largely dependent upon the cytosolic calcium concentration in intact cells [29]. On the other hand, cytosolic HBx triggers the release of calcium ions from intracellular stores and eventually enhances HBV replication [30]. Based on these results, we investigated whether HBx acts on the free intracellular calcium level to activate the CaMKK/AMPK pathway under glucose deprivation. The calcium-binding dye fluo-3 AM was used to evaluate intracellular calcium levels, and fluorescence was measured by flow cytometry. As shown in Fig. 6A, HBx transfection increased fluorescence intensity in hepatoma cells when exposed to glucose deprivation, whereas downregulation of HBx reduced the intracellular calcium concentration (Fig. 6B). Since HBx targets mainly mitochondrial calcium channels [30], HBx-transfected cells were treated with BAPTA-AM (a cytosolic calcium blocker) or cyclosporin A (CsA, a mitochondrial calcium channel blocker) to disrupt mitochondrial calcium signaling [31]. Under conditions of glucose limitation, BAPTA-AM or CsA treatment prevented HBx-induced phosphorylation of AMPK and ACC (Fig. 6C, 6D). This inhibitory effect was also observed in HBV-infected HepG2.2.15 cells in a concentration-dependent manner (Fig. 6E), implying that HBx-mediated mitochondrial calcium channels are required for activation of the AMPK pathway under such stressful conditions. Next, we determined whether HBx modulates AMPK activity in a cytosolic calcium/CaMKK-dependent manner. HBx-expressing cells were starved in medium without glucose and stimulated with STO-609, a CaMKK inhibitor [32]. As shown in Fig. 6F, STO-609 treatment had no significant impact on AMPK pathway activity in SMMC-7721 HBx cells cultured in complete medium. Under glucose deprivation, however, this inhibitor blocked HBx-induced AMPK and ACC phosphorylation in HBx-transfected hepatoma cells (Fig. 6F, 6G). Collectively, these data suggest that HBx stimulates the AMPK pathway by triggering the mobilization of intracellular calcium ions and activating calcium-responsive CaMKK in the absence of glucose.

### Inhibition of FAO reverses the HBx-mediated tumor growth advantage *in vivo*

**Figure 7:**
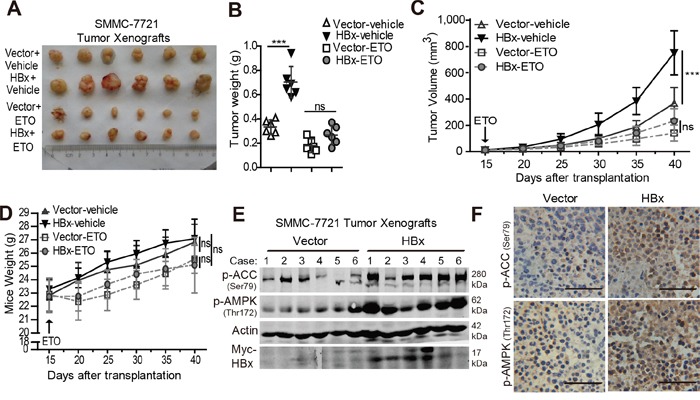
Overexpression of HBx enhances tumorigenic potential while inhibition of FAO reversed the tumor growth advantage *in vivo* **A.** Representative subcutaneous tumors from mice implanted with 1×10^6^ control or HBx-expressing cells treated with saline vehicle or 40mg/kg ETO for 5 weeks were shown. **B.** Tumor weights were measured when the mice were sacrificed (***p<0.001). Tumor growth progression **C.** and mice body weight **D.** from each group (n=6 per group) during the experimental treatments were evaluated at the indicated time points and data are shown as mean ± SD. (***p<0.001). **E.** Immunoblotting analyses of p-AMPK (Thr172), p-ACC (Ser79) and Myc-Tag (HBx) in subcutaneous tumors from mice implanted with vector control or HBx-expressing cells, β-actin was used as internal loading control. **F.** Immunohistochemical analyses of p-AMPK (Thr172) and p-ACC (Ser79) in Vector or HBx-expressing xenografts, and the representative photographs were shown (400x magnifications, Scale bar 50 μm).

Next, we determined whether HBx promotes HCC cell survival and enhances tumor formation by activating FAO *in vivo*. To address these issues, we performed xenograft assays using SMMC-7721 cells expressing a vector control or HBx. As shown in Fig. 7A and 7B, HBx expression significantly enhanced tumor formation *in vivo*, as revealed by the tumor growth curves and xenograft weights. Notably, inhibition of FAO by ETO markedly reduced the tumor burden in both groups. More importantly, no significant differences in tumor volume or weight were observed between the two ETO-treated groups at the end of the assay (Fig. 7C). Additionally, all mice were healthy and no obvious body weight loss or toxicity was observed over the 3 weeks of ETO treatment (Fig. 7D). These data suggest that FAO inhibition by ETO impairs the tumorigenic capacity of HBx *in vivo*. Furthermore, immunoblot and immunohistochemical analyses showed that overexpression of HBx enhanced the phosphorylation level of AMPK and ACC in the tumor xenografts (Fig. 7E, 7F). Taken together, these data demonstrate that HBx confers survival and growth advantage in tumor cells by activating the AMPK/ACC pathway and FAO during tumorigenesis *in vivo*, and interfering with FAO might represent an efficient strategy to overcome the tumor aggressiveness induced by HBx expression.

### HBx expression levels are positively correlated with activation of the AMPK signaling pathway and FAO in clinical HCC specimens

**Figure 8:**
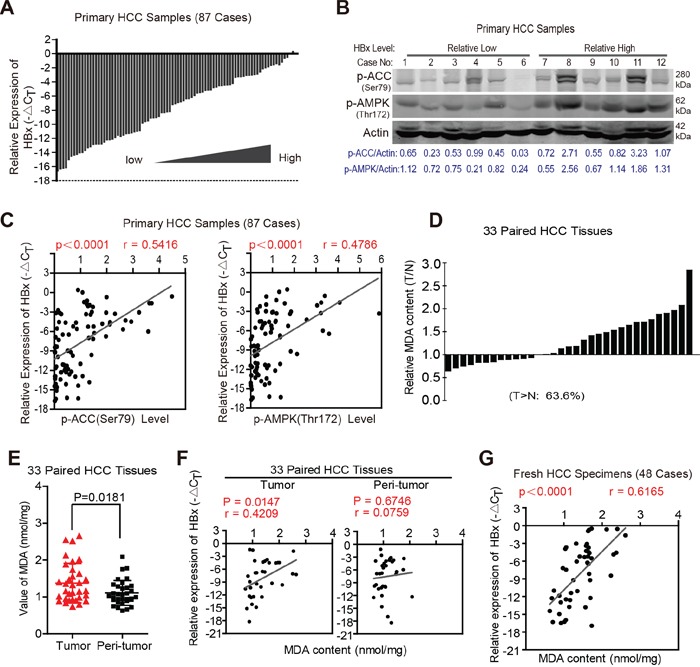
HBx expression levels are positively correlated with activation of AMPK signaling pathway and FAO in clinical HCC specimens **A.** 87 cases of human HCC specimens were collected and HBx mRNA expression levels were determined by RT-PCR. The level of actin mRNA was used as an internal control. **B.** 87 samples were lysed to determine the phosphorylation state of AMPK and its downstream target ACC by immunoblotting analyses, β-actin was used as internal loading control. The representative results of 12 cases were shown. **C.** The correlation analyses revealed that HBx expression levels were associated with the phosphorylation state of AMPK and ACC in 87 cases of human HCC specimens. **D.** Relative MDA content in 33 paired primary HCC tissue samples. (T/N>1: 21 cases of total 33 cases, 63.6%). **E.** Tumor samples exhibited higher MDA content than that in their peri-tumoral tissues. **F.** The positive correlation between HBx expression and MDA content was observed in 33 cases of HCC samples but not in their peri-tumoral tissues. **G.** The correlation analyses revealed the relevance between HBx expression and MDA content in another 48 fresh HCC specimens. Experiments were performed in triplicate and data were shown as mean ± SD.

Based on our findings in xenograft models *in vivo*, we further determined whether HBx expression was positively associated with the degree of AMPK activation and ACC phosphorylation in clinical HCC specimens. To address this issue, we first collected 87 human HCC specimens and detected the HBx expression levels by RT-PCR. As shown in Fig. 8A, the HBx expression in these specimens varied markedly. We next determined the phosphorylation state of AMPK and ACC in these samples (Fig. 8B and Supplementary Fig. 4). Notably, HBx expression levels were significantly correlated with the phosphorylation state of AMPK and ACC in human HCC samples (Pearson's coefficient, 0.542 and 0.479, respectively) (Fig. 8C). Because activation of the AMPK pathway facilitates FAO, we next explored the relationship between HBx and FAO in clinical HCC tissues. As malondialdehyde (MDA) is the end product of lipid catabolism via fatty acid β-oxidation [33, 34], we assessed the MDA contents and HBx mRNA expression levels in 33 paired HCC tissues. As shown in Fig. 8D and 8E, tumor samples exhibited higher MDA contents than did peritumoral tissues (average MDA contents: 1.375±0.54 vs. 1.108±0.3369; P=0.0181). More importantly, the MDA contents were positively associated with HBx levels in HCC samples but not in peritumoral tissues (Pearson's coefficient: 0.4209 vs. 0.0759; P-value: 0.0147 vs. 0.6747) (Fig. 8F). In addition, the results of an additional 48 fresh HCC specimens also revealed a positive correlation between HBx expression and MDA content (Fig. 8G), confirming the relationship between HBx expression and activation of FAO in clinical HCC tissues. Together, these results suggest a critical role for HBx in providing tumor cells a survival advantage during metabolic stress by activating the AMPK pathway and FAO *in vivo*.

## DISCUSSION

Glucose limitation can lead to energy deficiency and oxidative stress by decreasing intracellular ATP and NADPH contents. In the present study, we observed that overexpression of HBx prolonged hepatoma cell survival in response to low glucose. Along with this protective effect, we also found that HBx is able to prevent the glucose depletion-induced decrease in ATP and NADPH contents. Furthermore, overexpression of HBx increased activation of the AMPK pathway and FAO under glucose limitation, thus supplying energy for cell survival. Consistently, clinical HCC tissues with high HBx expression also exhibited enhanced activity of the AMPK pathway and elevated FAO. Therefore, HBx-expressing HCC cells may gain a survival benefit by inducing lipid catabolism to respond to environmental stress both *in vitro* and *in vivo*.

Accumulating evidence indicates that aberrant activation of lipid metabolism contributes to tumor formation [13]. HBx has been implicated in aberrant lipogenesis in HBV-related HCC. It was shown to upregulate the expression of lipogenesis and adipogenesis-associated enzymes and induce the accumulation of LDs [35]. Various cellular stresses stimulate the generation of LDs [36], which in turn fuel FAO to sustain cell viability [23]. Similarly, LD formation was demonstrated to attenuate ischemia-induced injury in the heart and promote neuron survival upon starvation [37, 38]. In addition to its role in promoting lipid accumulation, we found that HBx activated FAO upon glucose withdrawal. As nutrient starvation frequently occurs during solid tumor formation, HBx likely exerts a biphasic effect on intracellular lipid metabolism. On the one hand, it accelerates lipid synthesis and storage under standard culture conditions, while on the other hand, it facilitates mobilization and oxidation of lipids in stores to meet the energy demand caused by exposure to stressful conditions. Thus, this dynamic equilibrium of lipid metabolism is critical for survival of HBx-transformed tumor cells and warrants further investigation.

In response to glucose limitation, the highly glycolytic HCC cells are unable to switch their metabolic phenotype from glycolysis to FAO as an alternative source of biogenetic substrates. Eventually, this leads to energy crash and cell death. In this scenario, pharmacologic activation of FAO by BEZA could render HCC cells more resistant to glucose deprivation by shifting from a dependence on glycolysis to lipid metabolism. It also enables HCC cells to use fatty acids as fuel as a pro-survival adaptation. Interestingly, HBx functioned as an FAO activator during glucose deficiency to enhance FAO, thus providing catabolic substrates for OXPHOS. In accordance, inhibition of FAO by ETO reversed the HBx-mediated protective effects upon glucose withdrawal, underlying the importance of FAO activation for HBx-mediated enhancement of cell survival under metabolic stress.

It is widely accepted that oncogenes such as c-MYC and K-ras [9, 39, 40] or tumor suppressors (P53, PTEN) [41, 42] are involved in the re-programming of cellular metabolism once activated or inactivated. Of note, unlike these oncogenes, HBx expression did not affect glycolysis or OXPHOS in HCC cells under standard culture conditions. Instead, HBx-mediated activation of FAO occurred only during glucose depletion to protect against cell death. AMPK has been described as a sensor of cellular energy homeostasis under metabolic stress conditions [42]. Its activation is intimately linked with enhanced FAO. Herein, HBx-expressing cells displayed a higher degree of phosphorylation of AMPK and ACC than their counterparts in the absence of glucose. Moreover, HBx expression was positively correlated with activation of the AMPK and FAO pathways in human HCC tissues. Mechanistically, we focused on the upstream components of the AMPK signaling pathway. Liver kinase B1 (LKB1) and CaMKK are involved in the AMPK pathway [29, 43]. Since CaMKK is activated by calcium/calmodulin binding, we determined intracellular calcium levels. Interestingly, the mobilization of calcium ions from internal stores into the cytosol was facilitated by HBx expression during glucose deprivation. Indeed, overexpression of HBx resulted in transactivation of both the JNK and MAPK transduction pathways in association with release of cytosolic calcium [44]. However, further studies are required to elucidate the mechanism by which HBx upregulates the cytosolic calcium level under glucose limitation.

Given that its inactivation is involved in tumorigenesis [42, 45], AMPK is increasingly regarded as a promising therapeutic target for cancer treatment. For instance, pharmacologic activators of AMPK such as metformin and phenformin have been shown to delay or suppress tumor progression in several tumor xenograft models [46-48]. However, in our model, HBx-induced activation of the AMPK pathway and FAO promoted HCC cell survival under glucose deprivation. Therefore, we further suggest that AMPK agonists may be used in combination with FAO inhibitors to suppress tumor progression, particularly in HBV-associated HCCs in which metabolic stress frequently occurs [49].

In summary, our results demonstrate that HBx-induced FAO activation provides catabolic substrates for HCC cells to overcome glucose starvation, and pharmacological inhibitors of FAO such as etomoxir could represent an effective adjuvant anticancer treatment for HBV-related HCC.

## MATERIALS AND METHODS

### Human HCC specimens

All the fresh human tissue specimens used in this study were obtained from patients who underwent curative surgery for HCC at the Eastern Hepatobiliary Surgery Hospital (EHBH) in Shanghai, China. The procedure of human sample collection and analysis was approved by the Ethics Committee of EHBH.

### Reagents

Etomoxir (ETO) and STO-609 was purchased from TOCRIS (Tocris Bioscience, UK). Bezafibrate (BEZA), CCCP, and H_2_O_2_ were obtained from Sigma (St. Louis, MO, USA). Oligomycin was purchased from Cell Signaling Technology (USA). cyclosporin A (CsA) was obtained from Selleck Chemicals (USA) and BAPTA-AM was purchased from MedChemexpress Co. (USA). The calcium-sensitive indicator, Fluo-3 AM was purchased from Beyotime Co. (China). XF Palmitate-BSA FAO Substrate was obtained from seahorse Bioscience.

### Cell lines, cell culture and treatment

The human liver cancer cell lines SMMC-7721, Huh7, HepG2, and HepG2.2.15 (a HBV-transfected HepG2 cell line) were purchased from Cell Bank of Type Culture Collection of the Chinese Academy of Sciences (Shanghai Institute of Cell Biology). Cell lines were routinely cultured in Dulbecco's modifid Eagle's medium (with 4.5 g/L glucose and 2 mM glutamine) (Gibco) supplemented with 10% FBS (Gibco) within a humidified incubator containing 5% CO2 at 37°C. For the nutrient starvation experiments, hepatoma cells were washed with PBS twice and maintained in glucose-free DMEM (Gibco) containing 10% FBS, completed DMEM without FBS, or glutamine-free DMEM with 10% FBS, respectively.

### Gene silencing and plasmid transfection

Synthetic small interfering RNA (siRNA; scrambled and HBx-targeting siRNA) were purchased from Biotend Co. (Shanghai, China). The sequences of siRNA against HBx and negative control siRNA are shown as follow: siHBx #1 forward: GCCACAACGUCUAUAUCAUdTdT; reverse: AUGAUAUAGACGUUGUGGCdTdT; siHBx #2 forward: GGCAGAGGAAGUCUUCUAAdTdT; reverse: UUAGAAGACUUCCUCUGCCdTdT. The transfection of siRNAs was performed according to the manufacturers’ instructions. Briefly, SMMC-7721 or Huh7 HBx-expressing cells were cultured in 12-well for 12h and then were transfected with siRNA using lipofectamine 2000 reagent (Invitrogen, Carlsbad, CA). For the plasmid construction, the full-length encoding region of human HBx cDNA was subcloned into an Xho I/Bgl II site of a transposon-based PB-Myc-tagged vector. SMMC-7721 or Huh7 cells were plated in 12-well culture plates for 24h and then transfected with plasmids containing empty vector or PB-Myc-HBx constructs together with transposase encoding vector using jet-PEI (Polyplus, New York, NY) according to the manufacturer's instructions. At 48h after transfection, cells were replated in 6-well plates and selected with 0.5-1μg/ml puromycin. Following selection for 2 weeks, total sorted subsets of Puromycin-resistant cells were maintained under normal cultural medium and expanded into stable cell lines for the following experiments.

### Western blotting

Whole cell extracts or 87 HCC tumor specimens were prepared in lysis buffer, and western blot analysis was performed as described previously [50]. Specific primary antibodies used were as follow: anti-AMPK, phospho-AMPK (Thr172), phospho-ACC (Ser79), and Myc-Tag (9B11) were purchased from Cell Signaling Technology (USA). Antibody against ACCα was obtained from proteintech and β-actin from Santa Cruz Biotechnology (Heidelberg, Germany). After incubating with the fluorescein-conjugated secondary antibody, the immunocomplexes were detected using an Odyssey fluorescence scanner (Li-Cor, Lincoln, NE).

### qRT-PCR

Total RNA of cultured cells or human HCC samples were extracted using TRIzol reagent (Invitrogen) according to the manufacturer's protocols. Real-time PCR analyses were performed using an ABI 7300 Fast Real-Time PCR System (Applied Biosystems, Foster City, CA) and SYBR Green PCR kit (Applied TaKaRa, Otsu, Shiga, Japan). The ΔCt method was used with actin as an endogenous control for normalization of the results. The following primers were purchased from Invitrogen: (1) β-actin: forward 5′-GACTACCTCATGAAGATC-3′, reverse 5′-GATCCACATCTGCTGGAA-3′; (2) HBx: forward 5′-TAGGCTGTGCTGCCAACTG-3′, reverse 5′-GGTCGTTGACATTGCTGAGAG-3′.

### Colony formation assay

For colonogenicity analysis, 600 viable indicated hepatoma cells were placed in six-well plates for 72h and maintained in complete medium or low-glucose (1.5mM) medium for 10 days. ETO (100μM) or BEZA (400μM) was added at the beginning of the culture period for 10 days. Colonies were fixed with methanol and stained with methylene blue, the number of colonies was scored and the representative pictures were photographed.

### *In vitro* competition assay

For *in vitro* competition assays, SMMC-7721 vector cells and cells stably co-expressing GFP and HBx were 1:1 mixed and seeded in 6-well plates at a density of 2×10^5^ cells per well for overnight, and then the medium was replaced with low-glucose (1.5mM) DMEM supplemented with 10% FBS, glutamine-deprivated DMEM supplemented with 10% FBS, and completed DMEM with no FBS, respectively. After incubation for 4 days, cells were trypsinized and collected as single-cell suspension. The percentage of GFP+ subsets in different treatment groups were determined by flow cytometry.

### Measurement of endogenous ROS level

The intracellular ROS levels were detected by labeling 2×10^5^ hepatoma cells with redox-sensitive probes CellRox (5μM) (Life Technologies) for 30min at 37°C. Then the cells were washed twice and resuspended in 0.2ml PBS. Fluorescence of labeled cells was analyzed by flow cytometry.

### Glucose and lactate measurements

Glucose and lactate contents in culture medium were evaluated using the BS-200 Chemistry Analyzer (Mindray, China) and EnzyChrom™ D-Lactate Assay Kit (Bioassay, CA, USA), respectively. Data were normalized to cell number in each well. For glucose uptake assays, cells were maintained under normal conditions for 24h and 10μM 2-NBDG (life technologies, USA) was added to the medium for 30 min in the dark at 37°C. After washed with PBS twice, labeled cells were collected as single-cell suspensions and the fluorenscence intensities were determined by flow cytometry.

### Cellular GSH, NADPH assays

The intracellular NADP+, and NADPH levels were determined using a NADP/NADPH Quantitation Colorimetric Kit (Biovision) according to the manufacturer instructions. The concentration of NADP+ was calculated by subtracting NADPH from total NADP content. For measurement of GSH content, a QuantiChrom™ Glutathione (GSH) Assay Kit (Bioassay) was used as recommended instructions.

### Metabolic assays

Oxygen consumption rate (OCR) and Extracellular acidification rate (ECAR) were evaluated using the Seahorse XF96 extracellular flux analyzer as previously described [51]. Briefly, 2×10^4^ SMMC-7721 vector or HBx cells per well were seeded overnight in XF96 well plates (Seahorse Bioscience, North Billerica, MA, USA) in serum-free culture medium. One hour before XF assay, cells were equilibrated with unbuffered DMEM and maintained in 37°C for PH stabilization. Analyses were performed both at basal conditions and after injection of OLI (1mg/ml), FCCP (1mM), Antimycin A (5mM) at indicated time points. Intracellular ATP levels were examined with an ATP Bioluminescence assay kit (Beyotime, China) according to manufacturer instructions. The concentrations of ATP level were normalized to protein content.

### Fatty acid oxidation assay

Cellular OCR was used to evaluate the capacity of fatty acid oxidation in real time using the Seahorse XF96 Extracellular Flux Analyzer. 2×10^4^ SMMC-7721 vector or HBx cells per well were seeded in triplicates in an XF96 well culture plate. Then the growth medium was replaced with substrate-limited DMEM supplemented with 0.5 mM glucose, 1 mM glutamine, 0.5 mM carnitine, 1% FBS and maintained overnight in 37°C. On the day of assay, the FAO assay KHB buffer (110 mM NaCl, 4.7 mM KCl, 2 mM MgSO_4_, 1.2 mM Na_2_HPO_4_) supplemented with 2.5 mM glucose, 0.5 mM carnitine and 5mM HEPES was exchanged and adjusted to pH 7.4 in 37°C incubator. To examine free fatty acid oxidation, BSA conjugated palmitate (Seahorse Bioscience) was added to a final concentration of 50 μM. Basal OCR of cells treated with Palmitate-BSA or BSA vehicle alone were measured.

### Cell survival and growth assay

The detection of cell survival rate and cell growth were performed with cholecystokinin-8 (CCK-8)(Dojindo, Japan) assay as previously described [52]. Briefly, 7000 viable cancer cells were seeded in triplicates in 96-well plates. For nutrient deprivation experiments, the cells were washed with PBS and maintained in 100μl different cultural medium following one night incubation. At indicated time points, each well was mixed with 10μl CCK-8 and incubated for additional 1h before the OD values were detected at an absorbance of 450nm using a microplate reader (Synergy HT, USA). The relative survival rate was given as a percent of the control value. For the assays of cell viability, cells were seeded in 12-well plate at a density of 1×10^5^ cells per well and treated with indicated reagents or not. The number of viable cells were counted using a CASY TT-cell Counter (Innovatis, Germany) after trypan blue staining.

### Immunohistochemical staining

The xenograft tumor slides were incubated with the following primary antibodies: phospho-AMPK (Thr172) (1:100), phospho-ACC (Ser79) (1:100) (Cell Signaling Technology, USA). Anti-rabbit peroxidase-conjugated secondary antibody (Santa Cruz Biotechnology) and diaminobenzidine colorimetric reagent solution purchased from Dako (Carpinteria, CA) was used. The staining process were according to standard methods. Assessment of the staining was performed using the Image-scop software (Media Cybernetics, Inc.) according to the staining intensities and the percentage of positively stained cells, as described [53].

### Measurement of intracellular calcium levels

For detection of intracellular free calcium concentrations, indicated hepatoma cells were treated with 5μM fluo-3 AM for 45 min at room temperature. The cells were washed with PBS and incubated for another 15 min in the absence of fluo-3 AM in order to de-esterify the dye. Then the cells were resuspended and the fluorenscence intensities were determined by flow cytometry.

### Measurement of MDA content

The MDA contents in HCC specimens was determined using the thiobarbituric acid-reactive substances (TBARS) methods. The MDA assay kit was purchased from Jian Cheng. Co. (Nanjing, China). Briefly, HCC tissues were homogenized in phosphate-buffered saline (5%) and MDA levels were spectrophotometrically measured at λ=450 nm according to the manufacturer's instructions.

### *In vivo* xenograft assay

6-8 weeks old male athymic mice were purchased from Chinese Science Academy (Shanghai, China). All animal experiments were performed according to the Second Military Medical University Animal Care Facility and the National Institutes of Health guidelines. Approximately 1×10^6^ SMMC-7721 cells expressing vector or HBx were suspended in 100μl of DMEM and Matrigel (BD Bio-sciences) (1:1), and injected subcutaneously into right flank of each mouse. At 2 weeks after xenotransplantation, mice implanted with SMMC-7721 vector or HBx were respectively randomized into two groups and treated as follows: etomoxir (ETO) (40mg/kg i.p. every other day for 3 weeks) or saline as untreated vehicle. The size of subcutaneous tumors and mice weight were recorded at the indicated time point.

### Statistical analysis

All the Values presented are expressed as mean±SD. After acquiring all data for histological parameters and *in vitro* assays, χ2 test and Student's t-test were applied to determine statistical significance. A value of p<0.05 was considered significant. Data analysis was performed by the SPSS software (version 16; SPSS).

## SUPPLEMENTARY FIGURES



## Abbreviations

HCC: hepatocellular carcinoma
HBx: Hepatitis B virus X protein
AMPK: AMP-activated protein kinase
ACC: acetyl-CoA carboxylase
FAO: fatty acid β-oxidation
ETO: etomoxir
BEZA: Bezafibrate
FBS: fetal bovine serum
ROS: reactive oxygen species
NADPH: nicotinamide adenine dinucleotide phosphate
GSH: glutathione
ATP: adenosine triphosphate
2-NBDG: 2-(N-(7-Nitrobenz-2-oxa-1,3-diazol-4-yl)Amino)-2-Deoxyglucose
CCCP: Carbonyl cyanide 3-chlorophenylhydrazone
MDA: malondialdehyde
OCR: Oxygen consumption rate
ECAR: Extracellular acidification rate
LDs: lipid droplets

## References

[1] Marrero JA (2006). Hepatocellular carcinoma. Current opinion in gastroenterology.

[2] Brechot C, Pourcel C, Louise A, Rain B, Tiollais P (1980). Presence of integrated hepatitis B virus DNA sequences in cellular DNA of human hepatocellular carcinoma. Nature.

[3] Benhenda S, Cougot D, Buendia MA, Neuveut C (2009). Hepatitis B virus X protein molecular functions and its role in virus life cycle and pathogenesis. Advances in cancer research.

[4] Feitelson MA, Duan LX (1997). Hepatitis B virus X antigen in the pathogenesis of chronic infections and the development of hepatocellular carcinoma. The American journal of pathology.

[5] Henkler FF, Koshy R (1996). Hepatitis B virus transcriptional activators: mechanisms and possible role in oncogenesis. Journal of viral hepatitis.

[6] Huang J, Kwong J, Sun EC, Liang TJ (1996). Proteasome complex as a potential cellular target of hepatitis B virus X protein. Journal of virology.

[7] Zheng DL, Zhang L, Cheng N, Xu X, Deng Q, Teng XM, Wang KS, Zhang X, Huang J, Han ZG (2009). Epigenetic modification induced by hepatitis B virus X protein via interaction with de novo DNA methyltransferase DNMT3A. Journal of hepatology.

[8] Dang CV (2012). Links between metabolism and cancer. Genes & development.

[9] Levine AJ, Puzio-Kuter AM (2010). The control of the metabolic switch in cancers by oncogenes and tumor suppressor genes. Science.

[10] Hanahan D, Weinberg RA (2011). Hallmarks of cancer: the next generation. Cell.

[11] Vander Heiden MG, Cantley LC, Thompson CB (2009). Understanding the Warburg effect: the metabolic requirements of cell proliferation. Science.

[12] Warburg O (1956). On the origin of cancer cells. Science.

[13] Currie E, Schulze A, Zechner R, Walther TC, Farese RV (2013). Cellular fatty acid metabolism and cancer. Cell metabolism.

[14] Schulze A, Harris AL (2012). How cancer metabolism is tuned for proliferation and vulnerable to disruption. Nature.

[15] Hsu PP, Sabatini DM (2008). Cancer cell metabolism: Warburg and beyond. Cell.

[16] Hirayama A, Kami K, Sugimoto M, Sugawara M, Toki N, Onozuka H, Kinoshita T, Saito N, Ochiai A, Tomita M, Esumi H, Soga T (2009). Quantitative metabolome profiling of colon and stomach cancer microenvironment by capillary electrophoresis time-of-flight mass spectrometry. Cancer research.

[17] Laderoute KR, Amin K, Calaoagan JM, Knapp M, Le T, Orduna J, Foretz M, Viollet B (2006). 5′-AMP-activated protein kinase (AMPK) is induced by low-oxygen and glucose deprivation conditions found in solid-tumor microenvironments. Molecular and cellular biology.

[18] Cairns RA, Harris IS, Mak TW (2011). Regulation of cancer cell metabolism. Nature reviews Cancer.

[19] Jeon SM, Chandel NS, Hay N (2012). AMPK regulates NADPH homeostasis to promote tumour cell survival during energy stress. Nature.

[20] Samudio I, Harmancey R, Fiegl M, Kantarjian H, Konopleva M, Korchin B, Kaluarachchi K, Bornmann W, Duvvuri S, Taegtmeyer H, Andreeff M (2010). Pharmacologic inhibition of fatty acid oxidation sensitizes human leukemia cells to apoptosis induction. The Journal of clinical investigation.

[21] Zaugg K, Yao Y, Reilly PT, Kannan K, Kiarash R, Mason J, Huang P, Sawyer SK, Fuerth B, Faubert B, Kalliomaki T, Elia A, Luo X (2011). Carnitine palmitoyltransferase 1C promotes cell survival and tumor growth under conditions of metabolic stress. Genes & development.

[22] Carracedo A, Weiss D, Leliaert AK, Bhasin M, de Boer VC, Laurent G, Adams AC, Sundvall M, Song SJ, Ito K, Finley LS, Egia A, Libermann T (2012). A metabolic prosurvival role for PML in breast cancer. The Journal of clinical investigation.

[23] Cabodevilla AG, Sanchez-Caballero L, Nintou E, Boiadjieva VG, Picatoste F, Gubern A, Claro E (2013). Cell survival during complete nutrient deprivation depends on lipid droplet-fueled beta-oxidation of fatty acids. The Journal of biological chemistry.

[24] Cui M, Xiao Z, Sun B, Wang Y, Zheng M, Ye L, Zhang X (2014). Involvement of cholesterol in hepatitis B virus X protein-induced abnormal lipid metabolism of hepatoma cells via up-regulating miR-205-targeted ACSL4. Biochemical and biophysical research communications.

[25] You X, Liu F, Zhang T, Li Y, Ye L, Zhang X (2013). Hepatitis B virus X protein upregulates oncogene Rab18 to result in the dysregulation of lipogenesis and proliferation of hepatoma cells. Carcinogenesis.

[26] Kim KH, Shin HJ, Kim K, Choi HM, Rhee SH, Moon HB, Kim HH, Yang US, Yu DY, Cheong J (2007). Hepatitis B virus X protein induces hepatic steatosis via transcriptional activation of SREBP1 and PPARgamma. Gastroenterology.

[27] Kim JY, Song EH, Lee HJ, Oh YK, Choi KH, Yu DY, Park SI, Seong JK, Kim WH (2010). HBx-induced hepatic steatosis and apoptosis are regulated by TNFR1- and NF-kappaB-dependent pathways. Journal of molecular biology.

[28] Bungard D, Fuerth BJ, Zeng PY, Faubert B, Maas NL, Viollet B, Carling D, Thompson CB, Jones RG, Berger SL (2010). Signaling kinase AMPK activates stress-promoted transcription via histone H2B phosphorylation. Science.

[29] Hurley RL, Anderson KA, Franzone JM, Kemp BE, Means AR, Witters LA (2005). The Ca2+/calmodulin-dependent protein kinase kinases are AMP-activated protein kinase kinases. The Journal of biological chemistry.

[30] Bouchard MJ, Wang LH, Schneider RJ (2001). Calcium signaling by HBx protein in hepatitis B virus DNA replication. Science.

[31] Clapham DE (1995). Calcium signaling. Cell.

[32] Tokumitsu H, Inuzuka H, Ishikawa Y, Ikeda M, Saji I, Kobayashi R (2002). STO-609, a specific inhibitor of the Ca(2+)/calmodulin-dependent protein kinase kinase. The Journal of biological chemistry.

[33] Liu L, Zhang J, Li M, Zhang X, Li Z, Wang L, Wu J, Luo C (2014). Inhibition of HepG2 cell proliferation by ursolic acid and polysaccharides via the downregulation of cyclooxygenase-2. Molecular medicine reports.

[34] Li JL, Wang QY, Luan HY, Kang ZC, Wang CB (2012). Effects of L-carnitine against oxidative stress in human hepatocytes: involvement of peroxisome proliferator-activated receptor alpha. Journal of biomedical science.

[35] Na TY, Shin YK, Roh KJ, Kang SA, Hong I, Oh SJ, Seong JK, Park CK, Choi YL, Lee MO (2009). Liver X receptor mediates hepatitis B virus X protein-induced lipogenesis in hepatitis B virus-associated hepatocellular carcinoma. Hepatology.

[36] Boren J, Brindle KM (2012). Apoptosis-induced mitochondrial dysfunction causes cytoplasmic lipid droplet formation. Cell death and differentiation.

[37] Du L, Hickey RW, Bayir H, Watkins SC, Tyurin VA, Guo F, Kochanek PM, Jenkins LW, Ren J, Gibson G, Chu CT, Kagan VE, Clark RS (2009). Starving neurons show sex difference in autophagy. The Journal of biological chemistry.

[38] Lei P, Baysa A, Nebb HI, Valen G, Skomedal T, Osnes JB, Yang Z, Haugen F (2013). Activation of Liver X receptors in the heart leads to accumulation of intracellular lipids and attenuation of ischemia-reperfusion injury. Basic research in cardiology.

[39] Zwaans BM, Lombard DB (2014). Interplay between sirtuins, MYC and hypoxia-inducible factor in cancer-associated metabolic reprogramming. Disease models & mechanisms.

[40] Yun J, Rago C, Cheong I, Pagliarini R, Angenendt P, Rajagopalan H, Schmidt K, Willson JK, Markowitz S, Zhou S, Diaz LA, Velculescu VE, Lengauer C, Kinzler KW, Vogelstein B, Papadopoulos N (2009). Glucose deprivation contributes to the development of KRAS pathway mutations in tumor cells. Science.

[41] Bensaad K, Tsuruta A, Selak MA, Vidal MN, Nakano K, Bartrons R, Gottlieb E, Vousden KH (2006). TIGAR, a p53-inducible regulator of glycolysis and apoptosis. Cell.

[42] Shackelford DB, Shaw RJ (2009). The LKB1-AMPK pathway: metabolism and growth control in tumour suppression. Nature reviews Cancer.

[43] Hong SP, Momcilovic M, Carlson M (2005). Function of mammalian LKB1 and Ca2+/calmodulin-dependent protein kinase kinase alpha as Snf1-activating kinases in yeast. The Journal of biological chemistry.

[44] Oh JC, Jeong DL, Kim IK, Oh SH (2003). Activation of calcium signaling by hepatitis B virus-X protein in liver cells. Experimental & molecular medicine.

[45] Faubert B, Boily G, Izreig S, Griss T, Samborska B, Dong Z, Dupuy F, Chambers C, Fuerth BJ, Viollet B, Mamer OA, Avizonis D, DeBerardinis RJ, Siegel PM, Jones RG (2013). AMPK is a negative regulator of the Warburg effect and suppresses tumor growth *in vivo*. Cell metabolism.

[46] Buzzai M, Jones RG, Amaravadi RK, Lum JJ, DeBerardinis RJ, Zhao F, Viollet B, Thompson CB (2007). Systemic treatment with the antidiabetic drug metformin selectively impairs p53-deficient tumor cell growth. Cancer research.

[47] Huang X, Wullschleger S, Shpiro N, McGuire VA, Sakamoto K, Woods YL, McBurnie W, Fleming S, Alessi DR (2008). Important role of the LKB1-AMPK pathway in suppressing tumorigenesis in PTEN-deficient mice. The Biochemical journal.

[48] Zheng L, Yang W, Wu F, Wang C, Yu L, Tang L, Qiu B, Li Y, Guo L, Wu M, Feng G, Zou D, Wang H (2013). Prognostic significance of AMPK activation and therapeutic effects of metformin in hepatocellular carcinoma. Clinical cancer research.

[49] Marra M, Sordelli IM, Lombardi A, Lamberti M, Tarantino L, Giudice A, Stiuso P, Abbruzzese A, Sperlongano R, Accardo M, Agresti M, Caraglia M, Sperlongano P (2011). Molecular targets and oxidative stress biomarkers in hepatocellular carcinoma: an overview. J Transl Med.

[50] Qian YW, Chen Y, Yang W, Fu J, Cao J, Ren YB, Zhu JJ, Su B, Luo T, Zhao XF, Dai RY, Li JJ, Sun W, Wu MC, Feng GS, Wang HY (2012). p28(GANK) prevents degradation of Oct4 and promotes expansion of tumor-initiating cells in hepatocarcinogenesis. Gastroenterology.

[51] Varum S, Rodrigues AS, Moura MB, Momcilovic O, Easley CAt, Ramalho-Santos J, Van Houten B, Schatten G (2011). Energy metabolism in human pluripotent stem cells and their differentiated counterparts. PloS one.

[52] Zhang JW, Zhang SS, Song JR, Sun K, Zong C, Zhao QD, Liu WT, Li R, Wu MC, Wei LX (2014). Autophagy inhibition switches low-dose camptothecin-induced premature senescence to apoptosis in human colorectal cancer cells. Biochemical pharmacology.

[53] Wang RY, Chen L, Chen HY, Hu L, Li L, Sun HY, Jiang F, Zhao J, Liu GM, Tang J, Chen CY, Yang YC, Chang YX (2013). MUC15 inhibits dimerization of EGFR and PI3K-AKT signaling and is associated with aggressive hepatocellular carcinomas in patients. Gastroenterology.

