# Supplementary material for: HBx regulates fatty acid oxidation to promote hepatocellular carcinoma survival during metabolic stress
Source: Oncotarget. 2016 Jan 4;7(6):6711–26. doi: 10.18632/oncotarget.6817 (PMC4872744; doi:10.18632/oncotarget.6817)
Supplement: Supplementary file 1 [file oncotarget-07-6711-s001.pdf]

SUPPLEMENTARY FIGURES

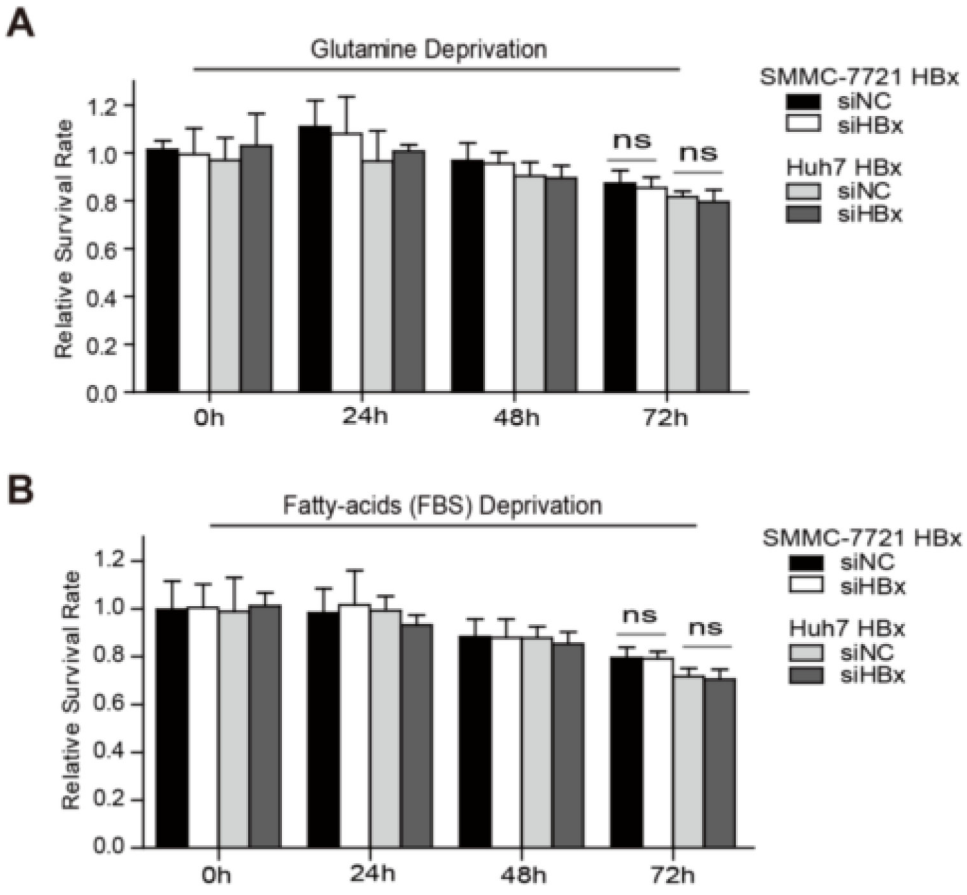

Supplementary Figure S1: HBx expression had no significant impact on cell survival under glutamine (-Gln) or FBS deprivation (-FBS). **A-B.** SMMC-7721-HBx and Huh7-HBx cells were transfected with siRNAs targeting HBx or negative control and cultured in medium without glutamine or FBS, respectively. The cell survival rates were measured at indicated time points. All the values were expressed as the fold change relative to their corresponding controls (presented as equal to 1) at the onset of the assays. Experiments were performed in triplicate and data were shown as mean ± SD.

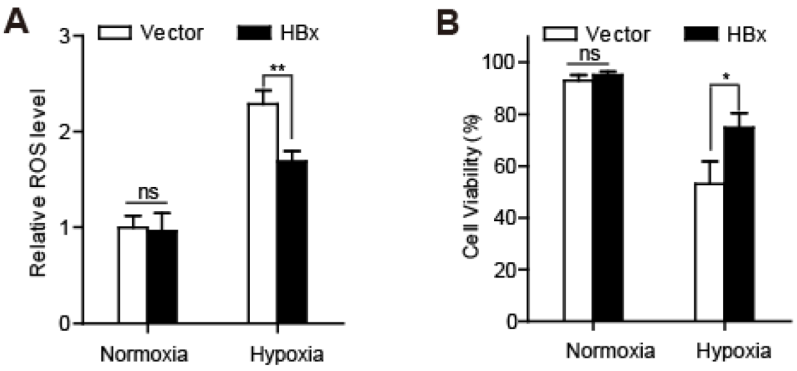

Supplementary Figure S2: A. Overexpression of HBx enhanced the elimination of intracellular ROS level induced by hypoxia treatment for 24h. All the values were expressed as the fold change relative to the untreated cells (presented as equal to 1). **B.** Overexpression of HBx promoted HCC cells survival upon oxidative stress caused by hypoxia. Experiments were performed in triplicate and data were shown as mean ± SD. (*p<0.05; **p<0.01).

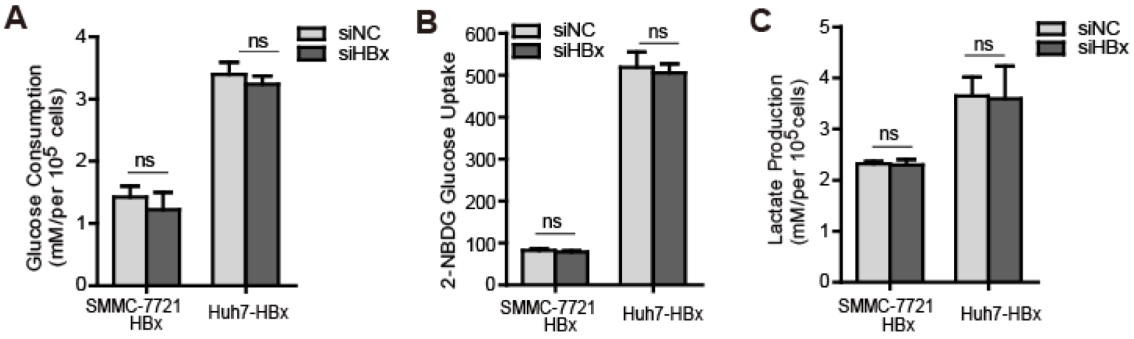

Supplementary Figure S3: Knockdown of HBx did not affect the glycolytic process in SMMC-7721-HBx and Huh7-HBx cells under standard cultural medium. SMMC-7721-HBx and Huh7-HBx cells were transfected with siRNA targeting HBx or negative control, the consumption of glucose **A.** and the production of lactate **C.** in medium were measured after maintained for 24h. All the values were normalized to cell number. **B.** The glucose uptake of hepatoma cells were detected via flow cytometry after incubation with 2-NBDG for 45 min. Experiments were performed in triplicate and data were shown as mean ± SD.

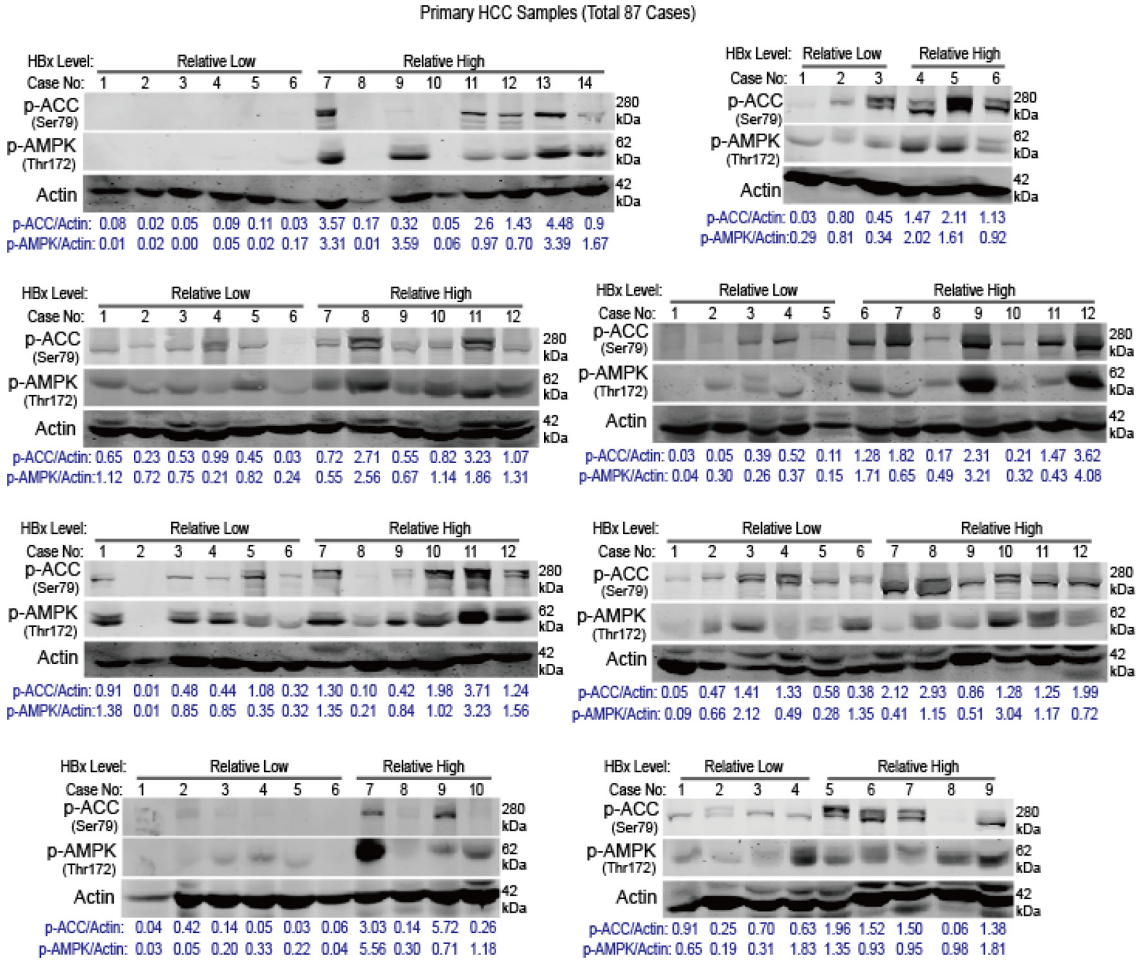

Supplementary Figure S4: 87 cases of human HCC specimens were collected and lysed to determine the phosphorylation state of AMPK and its downstream target ACC by immunoblotting analyses, β-actin was used as internal loading control.
